# Effect of conjugative transfer of antibiotic resistance genes mediated by plasmids on the microecology of different intestinal segments

**DOI:** 10.3389/fmicb.2024.1504659

**Published:** 2024-12-23

**Authors:** Chengshi Ding, Li Yan, Kai Zhang, Xiangxiang Liu, Ziyu Liu, Shaowei Hou, Jing Ma, Zhiping Wu, Hongfei Wei

**Affiliations:** ^1^College of Life Sciences, Zaozhuang University, Zaozhuang, China; ^2^General Practice, Tianjin Fifth Central Hospital, Tianjin, China; ^3^Key Laboratory for Synergistic Prevention of Water and Soil Environmental Pollution, Xinyang Normal University, Xinyang, China

**Keywords:** antibiotics resistance genes, conjugative transfer, different intestinal segments, plasmids, microecology

## Abstract

**Introduction:**

The conjugative transfer of antibiotic resistance genes (ARGs) mediated by plasmids occurred in different intestinal segments of mice was explored.

**Methods:**

The location of ARG donor bacteria and ARGs was investigated by qPCR, flow cytometry, and small animal imaging. The resistant microbiota was analyzed by *16S rRNA* gene amplification sequencing.

**Results:**

The small intestine was the main site for the location of ARG donor bacteria and ARGs. The intestinal microbiota richness of the small intestine (duodenum and jejunum) and the large intestine (cecum, colon, and rectum) increased, and the ileum microbiota richness decreased under the action of donor bacteria. The differences in the number of bacteria in the small intestine and the large intestine, as well as the relative richness of Firmicutes from the small intestine to the large intestine, decreased. By contrast, the relative abundance of Proteobacteria increased. The intake of resistant plasmids alleviated the impact of antibiotics on intestinal microbiota, particularly increasing the proportion of Proteobacteria and Bacteroides, which were presumed to be susceptible to ARGs.

**Discussion:**

The acquisition of ARGs by intestinal microbes is an important reason why infectious diseases are difficult to cure, which brings risks to human health and intestinal microecology.

## Introduction

In humans and other vertebrates, the gut microbial ecosystem is closely related to several basic host functions, including processing indigestible dietary components, defense against invasion by foreign organisms, and maturation of the immune system. The dynamic ecological balance of intestinal microbiota leads to physiological functions that are necessary for individual survival (Hooper et al., 2002; Nishida et al., 2018). The emergence and uncontrolled increase of antibiotic resistance leads to the dysregulation of intestinal microbiota and poses risks to the treatment of infectious diseases (Cantón, 2009; Aslam et al., 2018). Gram-negative bacteria, Gram-positive bacteria, aerobic bacteria, anaerobic bacteria, culturable bacteria, and unculturable bacteria can obtain antibiotic resistance, and antibiotic resistance genes (ARGs) are their genetic basis (Aminov and Mackie, 2007; de Niederhäusern et al., 2007; Denning et al., 2011; Pachori et al., 2019). At present, the location rules of ARGs in different intestinal segments, their effects on intestinal microbiota, and the susceptible microbiota of ARGs remain unclear. Thus, further studies must be conducted.

The intestines of vertebrates are colonized by a large number of diverse and complex microorganisms, which live in groups and are known as intestinal microbiota. Traditional studies on intestinal microorganisms are mostly based on *in vitro* culture, which can only reflect a small part of intestinal microorganisms, and many non-culturable microorganisms have been ignored. The development of *16S rRNA* gene amplification sequencing has increased our understanding of intestinal microbes. In addition, the rapidly developing second-generation high-throughput sequencing platform can quickly, conveniently, and accurately perform species annotation and abundance analysis of intestinal microbiota, thereby providing comprehensive understanding of the composition and distribution of vertebrate intestinal microbiota (Caporaso et al., 2011; Walters et al., 2015).

In this study, antibiotic resistant bacteria with the labeled genome of chloramphenicol resistance gene constructed in our laboratory and containing the conjugative transfer plasmid were used. Such bacteria were fed to mice to observe the location rule and transfer mechanism of resistant bacteria and ARGs in the intestines of mice. Mice were also fed with the constructed fluorescently labeled donor bacteria, and a fluorescence tracer was used to observe the location of ARGs in the intestines of mice. *16S rRNA* gene high-throughput sequencing was used to analyze the effects of antibiotic resistant bacteria on the species and distribution of intestinal microorganisms. The technology provided us comprehensive understanding of the intestinal microbiota involved in conjugative transfer and determines which intestinal microbes likely accept resistant plasmids and become new resistant bacteria.

## Materials and methods

### Strains, plasmids and resistance genes

All donor bacteria were constructed from wild-type *Escherichia coli* K12 MG1655 (ATCC 47076) with artificial genetic modification. Conjugative plasmid RP4 is a multidrug-resistant plasmid (60,099 kb), which is capable of spontaneous transfer, stable survival and replication in multiple host cells, belonging to the incompatibility P group (IncPα), carrying ampicillin (*Amp^R^*, *bla*), tetracycline (*Tet^R^*), and kanamycin (*Km^R^*) resistance genes. K12 (Cm: RP4) is a resistant bacterium constructed in our laboratory, which contains the plasmid RP4, and the *E. coli* genome was labeled with chloramphenicol resistance gene (Cm). The fluorescence-labeled donor bacterium K12Td-Tomato: RK2 constructed in our laboratory indicates that the genome of the resistant bacterium *E. coli* K12 is labeled with red fluorescent protein gene *Td-Tomato* and carries the conjugative plasmid RK2. The fluorescence-labeled donor bacterium K12: RK2 (an enhanced green fluorescent protein [EGFP]) constructed in our laboratory indicates that the plasmid RK2 of the resistant bacterium *E. coli* K12 is labeled with EGFP (Fu, 2017; Yang et al., 2019).

### Grouping and feeding of animals

Male Kunming mice (20 ± 2 g, Beijing Huafukang Biotechnology Co., Ltd., China) were randomly divided into six groups with 15 mice in each group, namely, the normal saline (NS) control group, antibiotic resistant bacteria (*E. coli* Cm: RP4) group, antibiotic group, antibiotic resistant bacteria (*E. coli* Cm: RP4) + low-dose antibiotic group, antibiotic resistant bacteria (*E. coli* Cm:RP4) + medium-dose antibiotic group, and antibiotic resistant bacteria (*E. coli* Cm: RP4) + high-dose antibiotic group. All mice were fed adaptively for 3 days in a specific pathogen-free animal laboratory. Each experimental group was placed in a separate room. In the NS control group, each mouse was given 1 mL of NS per day for 2 consecutive days, with free intake of sterile distilled water and artificial rat food. The composition of the artificial rat food included corn, soybean meal, flour, wheat bran, fish meal, meat and bone meal, etc. (water ≤ 100 g/kg, crude protein ≥ 200 g/kg, crude fat ≥ 40 g/kg, crude fiber ≤ 50 g/kg). In the antibiotic resistant bacteria group, each mouse was given 1 mL of NS per day, containing 10^8^ cfu *E. coli* (Cm: RP4), and was given free access to sterile distilled water and artificial rat food for 2 consecutive days. In the antibiotic group, each mouse was given 1 mL of NS for 2 days and free access to 50 mg/L Tet, Km, Amp aqueous solution, and artificial rat food. In the antibiotic resistant bacteria + antibiotic group, each mouse was given 1 mL of NS containing 10^8^ cfu *E. coli* (Cm: RP4) per day for 2 consecutive days, and the mice freely consumed 25 mg/L (low dose), 50 mg/L (medium dose), 100 mg/L (high dose) Tet, Km, Amp aqueous solution and artificial food. NS, NS with resistant bacteria were administered by gavage. Sterile distilled water and antibiotic solutions were freely ingested through drinking water. This study was approved by the Ethics Committee of Zaozhuang University.

### Collection and disposal of animal feces

Fresh feces (0.11 g) were collected and placed in a sterile 2 mL centrifuge tube at 4 PM every day, added with 1.5 mL of NS, and crushed with sterile eye forceps. The mixture was mixed well to obtain a fecal suspension.

### Preparation of intestinal segments and intestinal microbes

After 16 days of feeding, the whole intestine was collected, and the outer surface of the intestine was washed with sterile NS. The small intestine was divided into the duodenum, jejunum, and ileum from front to back, and the large intestine was divided into the cecum, colon, and rectum. Different intestinal segments were cut into pieces, swirled at 500 rpm for 2 min, and centrifuged at 1,000 rpm for 10 s. Large chunks of tissue and food residue were discarded in the intestines. Then, the intestinal microbiota in the supernatant was collected by centrifugation at 5,000 rpm for 10 min, and the precipitate was washed two times with phosphate buffer (PBS, 0.01 mol/L, pH7.4, KGB5001, KEYGEN, China).

### Screening of resistant strains in the feces of NS control mice

Animal feeding was performed in accordance with the “Grouping and feeding of animals.” Animal feces were treated with bacterial suspension, washed two times with NS, and coated on a Luria–Bertani (LB) resistant agar plate, containing 50 mg/L Tet, 60 mg/L Km, and 80 mg/L Amp (Qiu et al., 2012; Ding et al., 2021). Overnight culture was performed at 37°C to observe the colonies.

### Detection of donor bacteria and resistant plasmid in feces

The feces were suspended with NS, and centrifuged at 2,000 × *g* for 10 min. Afterward, the precipitation was collected, and was washed two times with NS. Intracellular DNA was extracted using the Tiangen stool genomic DNA extraction kit (DP328, Tiangen Biotechnology Co., Ltd., Beijing, China). ARGs were detected by qPCR. Twenty microliters reaction system: 10 μL SYBR Green PCR Master Mix 2× (Roche, Basel, Switzerland), each primer 1 μL, 1 μL DNA template, 7 μL enzyme-free water. Two-step PCR reaction conditions: predenaturation, 95°C, 10 min; 95°C, 15 s, 60°C, 1 min, 40 cycles. The primers of resistance gene *Cm* were 5′-AGGCGGGCAAGAATGTGAATAAAG-3′ and 5′-ATCCCAATGGCATCGTAAAGAACA-3′, and the PCR amplification product was 159 bp. The primers of *traG* gene, which was the representative gene of antibiotic resistant plasmid RP4, were 5′-AAAGCGGACAGCATCAGTAACGAA-3′ and 5′-GAGCTTGGTGGCCGCATAGTGTAG-3′, and the PCR amplification product was 104 bp. The *16S rRNA* gene was used as the internal reference, the primers were 5′-CCTACGGGAGGCAGCAG-3′ and 5′-ATTACCGCGGCTGCTGG-3′, and the product was 194 bp. An absolute quantitative method equipped with the LightCycler 480II PCR instrument was used for qPCR (Roche, Basel, Switzerland). *Cm* and *traG* genes in feces were detected at days 1, 2, 4, 6, 8, 10, 12, and 14 after intragastric administration.

### Flow cytometry

Male Kunming mice were randomly divided into two groups: saline control group (NS group) and antibiotic resistant bacteria K12: RK2 (labeled with EGFP gene) group. In the antibiotic resistant bacteria group, each mouse was given 1 mL of NS per day, containing the antibiotic resistant bacteria K12: RK2 (EGFP), and was given free access to sterile 50 mg/L apramycin (Ap) aqueous solution and artificial rat food for 2 consecutive days. All other procedures were in accordance with the “Grouping and feeding of animals.” The feed was continuously administered for 1 week. Intestinal microbiota suspension was diluted 1,000 times (10^6^ cells/mL) by using upflow cytometry Becton Dickinson FACSCalibur (Becton, Dickinson and Company, United States). Flow screening was performed to determine the proportion of recipient bacteria (including green fluorescence).

### Small animal imaging

Male Kunming mice were randomly divided into four groups: saline control group (NS group), antibiotic resistant bacteria *E. coli* K12 (*Td-Tomato* and RK2) (labeled with *Td-Tomato* gene) group, antibiotic group, and antibiotic resistant bacteria K12 (*Td-Tomato* and RK2) + antibiotic group. In the antibiotic resistant bacteria group, each mouse was given 1 mL of NS per day, containing the antibiotic resistant bacteria K12 (*Td-Tomato* and RK2), and was given free access to sterile distilled water and artificial rat food for 2 consecutive days. In the antibiotic group, each mouse was given 1 mL of NS through intragastric administration for 2 days, and free access to sterile 50 mg/L Ap aqueous solution and artificial rat food. In the antibiotic resistant bacteria + antibiotic group, each mouse was gavage with bacteria K12 (*Td-Tomato* and RK2) at 1 mL NS per day for 2 consecutive days, with free ingestion of 50 mg/L Ap aqueous solution and artificial food. All other procedures were performed in accordance with the “Grouping and feeding of animals.” After feeding for a week, the mice were sacrificed; the whole intestine was taken, and the distribution of donor bacteria was observed by IVIS Lumina XR SeriesIII (Caliper Company, United States).

### Intestinal microbiota analysis

Mice were fed in accordance with the “Grouping and feeding of animals,” and the total DNA of intestinal and fecal bacteria in different parts of the antibiotic resistant bacteria + antibiotic group was extracted by using the Tiangen stool genome DNA kit, and identified by agar-gel electrophoresis. The V4 region of 16S rDNA was amplified using 27F (5′-AGAGTTTGATCCTGGCTCAG-3′) and 1492R (5′-TACCTTGTTACGACTT-3′) primers. Amplification was followed by high-throughput sequencing at the Illumina MiSeq PE250 second-generation sequencing platform (Novogene, Beijing, China). Uparse software v7.0.1001 was used to divide the sequences with 97% consistency into operational taxonomic units (OTUs). OTU clustering and species classification based on valid labels were performed. At the phylum level, the relative abundance and heat maps of intestinal microbiota in different parts of the intestine and feces were analyzed (Caporaso et al., 2011; Walters et al., 2015).

## Results

### Health of the mice

The mice in each group were healthy, and they did not die. In the antibiotic group and the antibiotic + resistant bacteria group, the diet of mice was reduced.

### Background of antibiotic resistance of intestinal microbes

**Figure 1:**
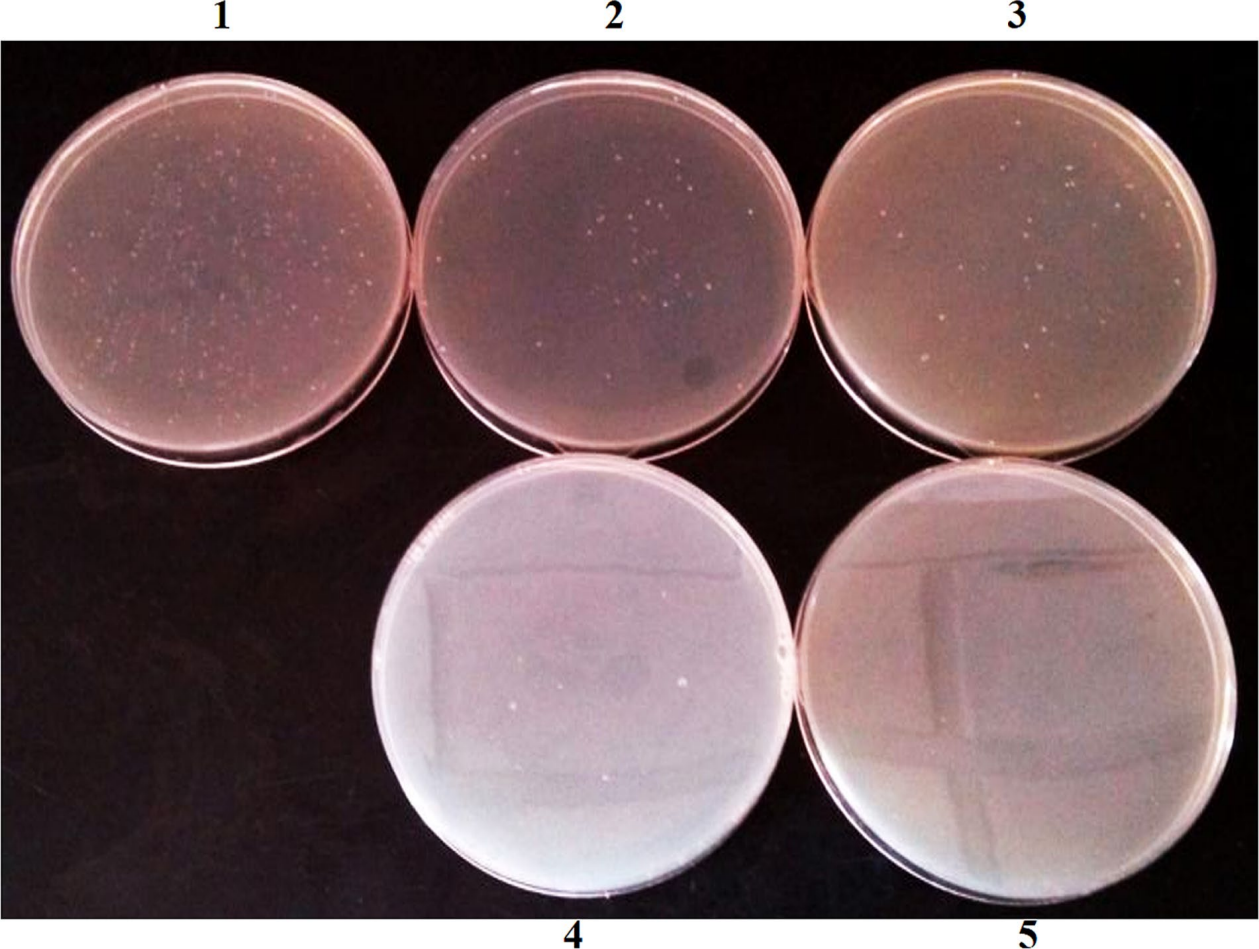
Antibiotic resistant bacteria isolated from mouse feces in NS control group. (1) No antibiotic; (2) Tet; (3) Km; (4) Amp; (5) Tet + Km + Amp.

The results showed that no culturable bacteria in the gut of normal mice could resist three antibiotics simultaneously, so we selected the conjugative transfer plasmid RP4 containing three genes that are resistant to Tet, Km, and Amp. In studying the location of resistant bacteria, the genome of resistant bacteria was labeled with the chloramphenicol resistance gene (*Cm*) (Figure 1).

### Distribution of plasmids (ARGs) in feces

**Figure 2:**
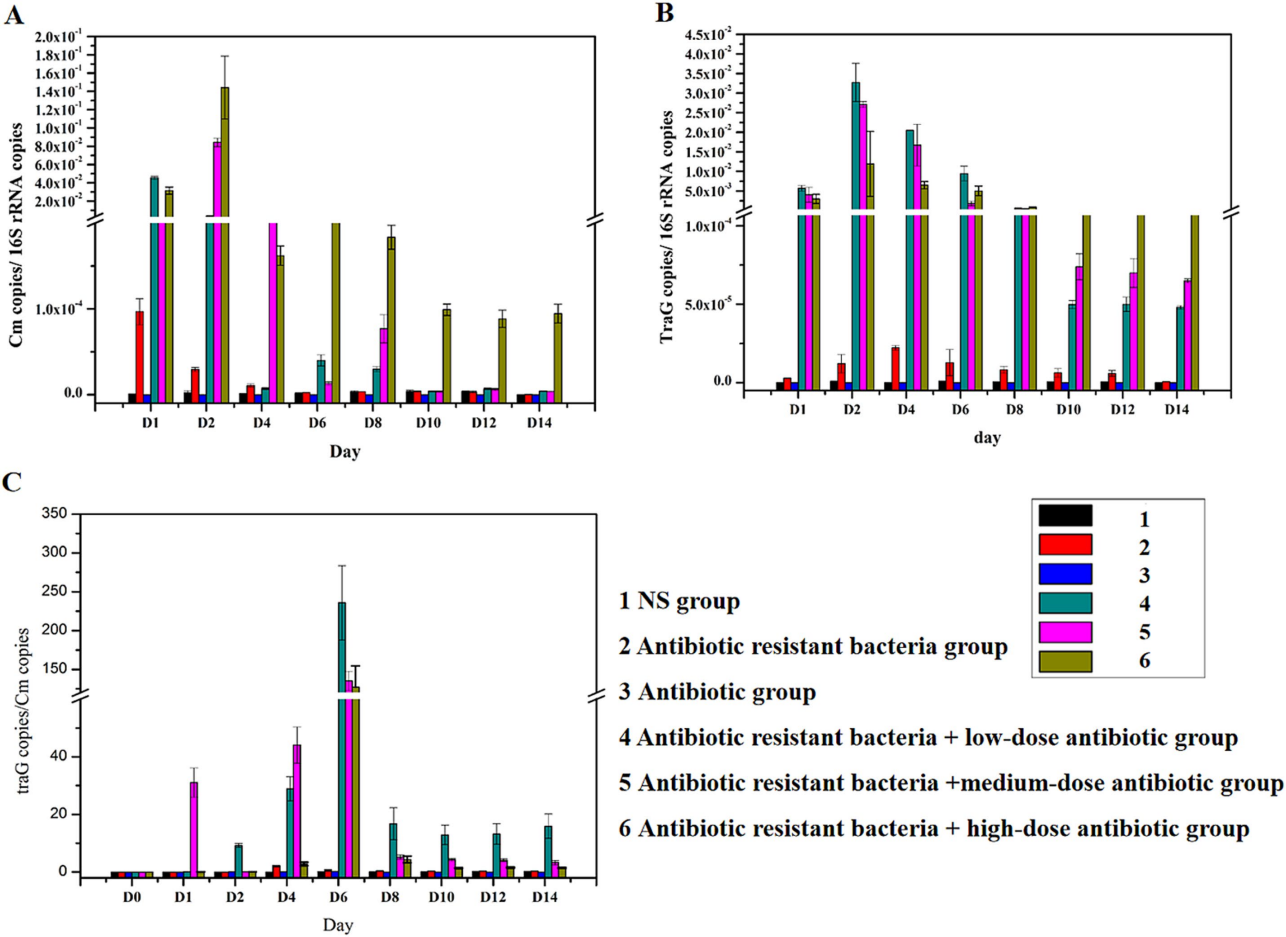
Distribution of plasmids (ARGs) in feces. **(A)** Distribution of the *Cm* gene; **(B)** distribution of *traG* gene; **(C)** changes in *traG* gene copies/*Cm* gene copies.

Feces were collected on days 1, 2, 4, 6, 8, 10, 12, and 14 after intragastric administration. In addition, bacterial DNA was extracted from feces, and the content of the *Cm* gene and *traG* gene (RP4) was detected. The amount of *16S rRNA* gene referred to the number of bacteria. The content of *Cm* gene in the genome represented the amount of RP4 donor bacteria. After inoculation, the amount of K12 (*Cm*: RP4) in feces initially increased (0–2 days), then decreased (2–10 days), and remained at a constant range after 10 days. Antibiotics increased the distribution of ARG donor bacteria (Figure 2A). The content of *traG* gene represented the amount of antibiotic resistant plasmid RP4. The amount of RP4 in feces increased initially (0–2 days), then decreased (2–10 days) after inoculation, and remained at a constant range after 10 days. Antibiotics increased the distribution of antibiotic resistant bacteria (Figure 2B). The *traG* gene copies/*Cm* gene copies represented the conjugative transfer of antibiotic resistant plasmid RP4. The ratio increased initially (0–6 days) and then decreased rapidly (6–8 days) in feces after inoculation, and remained in a constant range after 8 days. Antibiotics increased the ratio. From 2 to 6 days, RP4 continuously increased when *Cm* did not increase (antibiotic resistant donor bacteria did not increase), indicating that the conjugative transfer of ARGs occurred in the intestines of mice (Figure 2C).

### Distribution of ARGs in different intestinal regions

**Figure 3:**
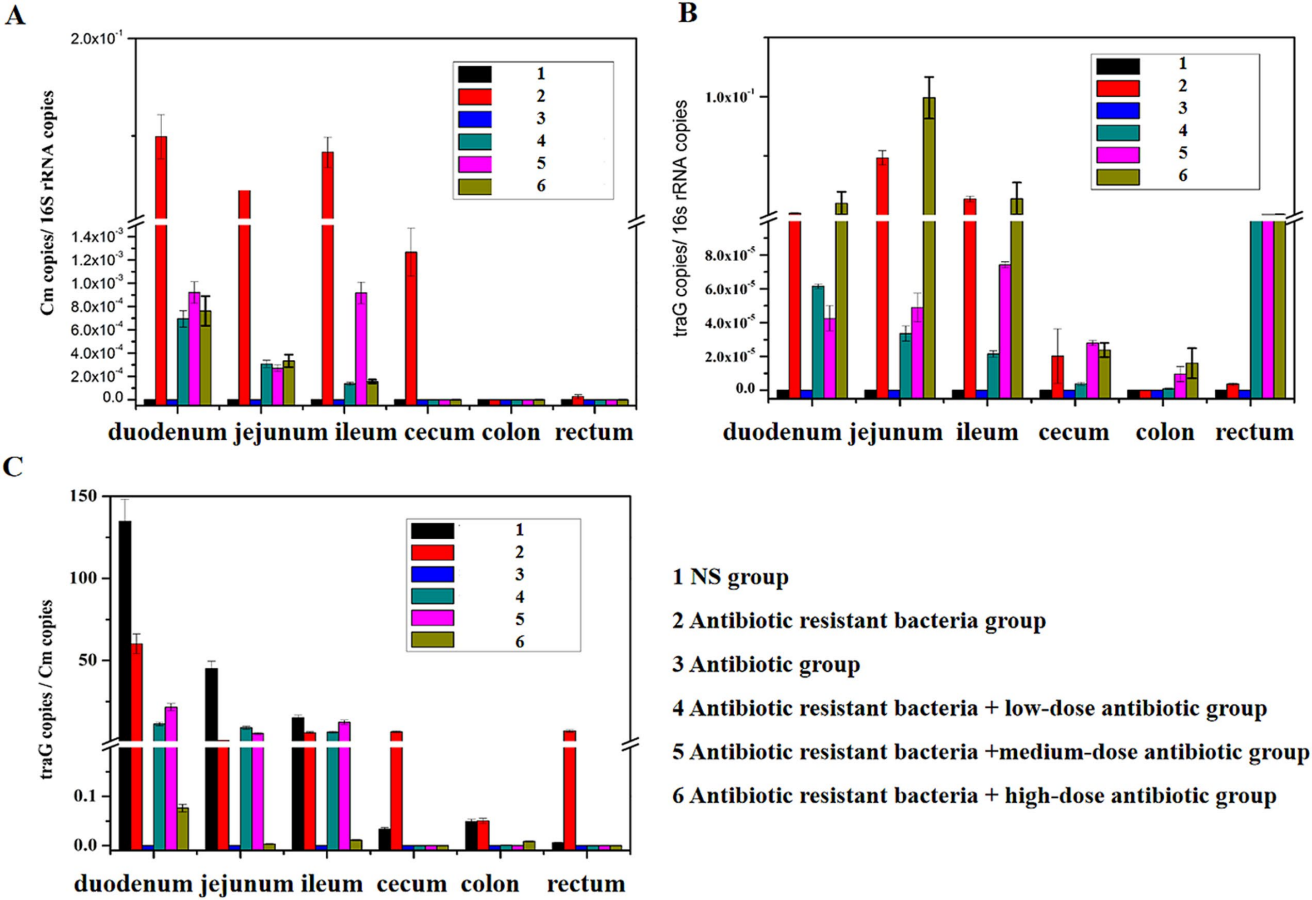
Distribution of ARGs in different intestinal regions. **(A)** Distribution of the *Cm* gene in different intestinal regions; **(B)** distribution of the *traG* gene in different intestinal regions; **(C)** changes of *traG* gene copies/*Cm* gene copies in different intestinal regions.

*Cm* gene copies/16S rRNA gene copies represented the amount of donor bacteria. TraG gene copies/16S rRNA gene copies represented the amount of plasmid RP4. TraG gene copies/*Cm* gene copies represented the amount of conjugative transfer. The donor bacteria were mainly distributed in the small intestine (duodenum, jejunum, and ileum), whereas few donor bacteria were found in the large intestine (cecum, colon, and rectum; Figure 3A). In the intestine, antibiotic resistant plasmid RP4 is mainly distributed in the small intestine (duodenum, jejunum, and ileum), whereas the antibiotic resistant plasmid RP4 was rarely found in the large intestine (cecum, colon, and rectum; Figure 3B). Compared with the distribution of bacterial quantity, the conjugative transfer of the antibiotic resistant plasmid RP4 primarily occurred in the small intestine (duodenum, jejunum, and ileum), whereas little conjugative transfer was observed in the large intestine (cecum, colon, and rectum; Figure 3C).

### Distribution of conjugants in the gut

In the flow cytometry experiment, as shown in Table 1 and Supplementary Figures S1, S2, the conjugants of antibiotic resistant bacteria (0.0148 ± 0.0012) in the small intestine (duodenum, jejunum, and ileum) were significantly more than that (0.0079 ± 0.0008) in the large intestine (cecum, colon, and rectum). So the small intestine was the main site for the transfer of ARGs.

**Table 1:** Ratio of conjugons (%).

Intestinal region	Ratio of conjugants (%)
Duodenum	0.0031 ± 0.0004
Jejunum	0.0034 ± 0.0003
Ileum	0.0083 ± 0.0007
Cecum	0.0022 ± 0.0002
Colon	0.0015 ± 0.0002
Rectum	0.0034 ± 0.0005

**Figure 4:**
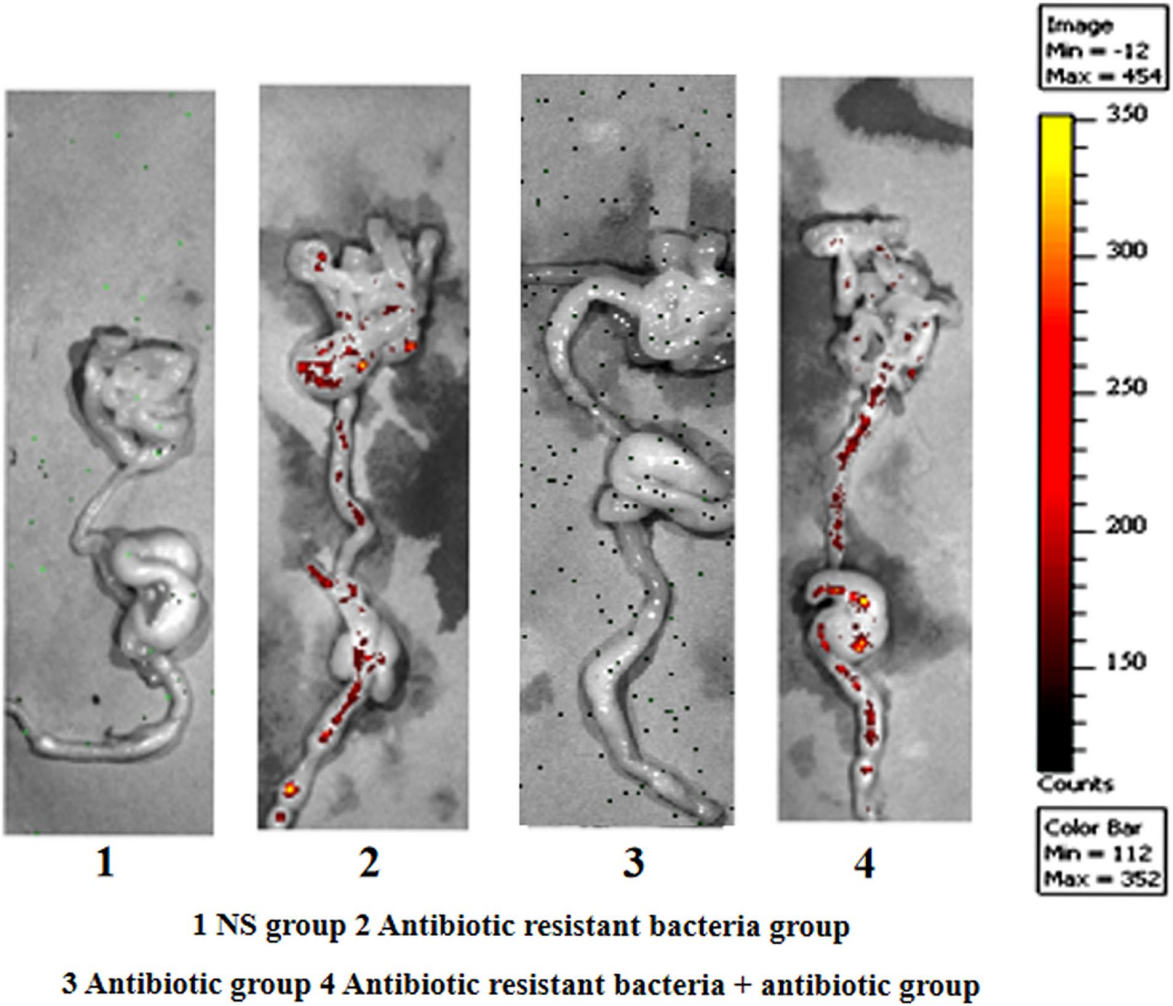
Imaging of small animals.

In the imaging experiment of small animals, with regard to absolute quantity, ARG donor bacteria in the control group were uniformly distributed in different parts of the intestine. Under the action of antibiotics, ARG donor bacteria were enriched in the distal ileum and large intestine in absolute quantity (Figure 4).

### Intestinal microbiota analysis

**Figure 5:**
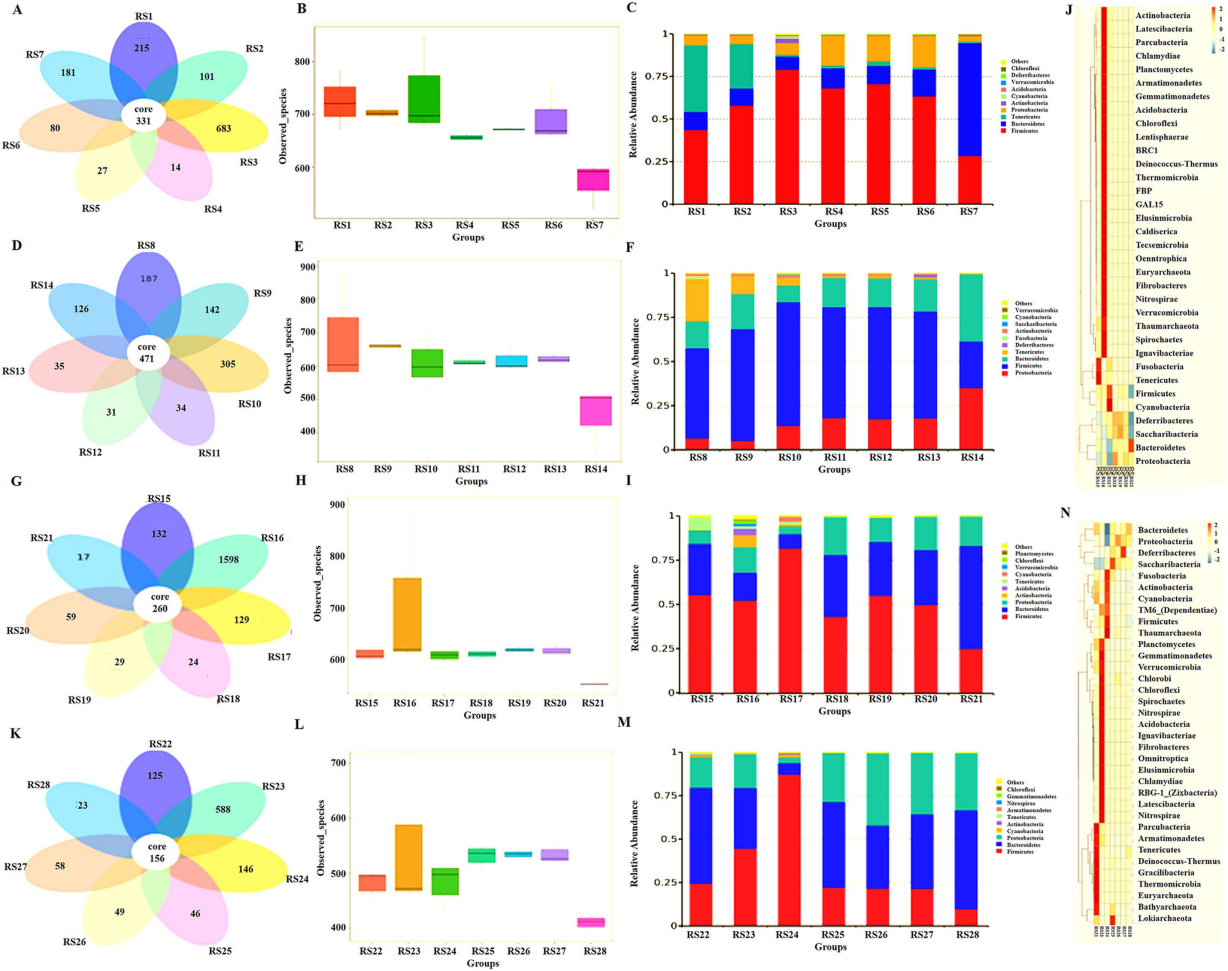
Intestinal microbiota analysis in different groups. **(A)** Abundance of intestinal microbiota in the NS control group, RS1 duodenum, RS2 jejunum, RS3 ileum, RS4 cecum, RS5 colon, RS6 rectum, and RS7 feces. **(B)** Number of species observed of intestinal microbiota in the NS control group. **(C)** Intestinal microbiota analysis at the phylum level in the NS control group. **(D)** Abundance of intestinal microbiota in the antibiotic resistant bacteria group, RS8 duodenum, RS9 jejunum, RS10 ileum, RS11 cecum, RS12 colon, RS13 rectum, and RS14 feces. **(E)** The number of species observed of intestinal microbiota in the antibiotic resistant bacteria group. **(F)** Intestinal microbiota analysis at the phylum level in the antibiotic resistant bacteria group. **(G)** Abundance of intestinal microbiota in antibiotic group, RS15 duodenum, RS16 jejunum, RS17 ileum, RS18 cecum, RS19 colon, RS20 rectum, and RS21 feces. **(H)** The number of species observed of intestinal microbiota in the antibiotic group. **(I)** Intestinal microbiota analysis at the phylum level in the antibiotic group. **(J)** The cluster heat map of intestinal microbiota in the antibiotic group. **(K)** Abundance of intestinal microbiota in the antibiotic resistant bacteria + medium-dose antibiotic group, RS22 duodenum, RS23 jejunum, RS24 ileum, RS25 cecum, RS26 colon, RS27 rectum, and RS28 feces. **(L)** The number of species observed of intestinal microbiota in the antibiotic resistant bacteria + medium-dose antibiotic group. **(M)** Intestinal microbiota analysis at the phylum level in the antibiotic resistant bacteria + medium-dose antibiotic group. **(N)** The cluster heat map of intestinal microbiota in the antibiotic resistant bacteria + medium-dose antibiotic group.

In the NS group, the OTUs of intestinal microbiota in the small intestine (duodenum, jejunum, and ileum) (999) and feces (181) were more than that (121) in the large intestine (cecum, colon, and rectum; Figure 5A). The number of species observed was similar in all parts of the small intestine (duodenum, jejunum, and ileum) and similar in all parts of the large intestine (cecum, colon, and rectum). The number of species in feces was different from that in the small intestine (Figure 5B). At the phylum level, Firmicutes, Bacteroidetes, Tenericutes, and Proteobacteria were the dominant bacteria in duodenum and jejunum, and the content of other bacteria was lower than 3%. Compared with the jejunum, the number of Firmicutes and Proteobacteria increased in the ileum, whereas Bacteroidetes and Tenericutes decreased. The number of Actinobacteria and Cyanobacteria also increased. The composition of intestinal microbiota in the cecum, colon, and rectum of the large intestine was similar, with Firmicutes, Bacteroidetes, and Proteobacteria as the dominant microbiota. The composition of microbiota in feces was also different from that in the large intestine, and Bacteroidetes was more evident (Figure 5C).

Compared with the NS group, the abundance of intestinal microbiota the jejunum and large intestine (cecum and colon) increased, whereas that in the duodenum and ileum decreased (Figure 5D). The difference in the number of observed microbiota decreased in the small and large intestines (Figure 5E). The relative abundance of Firmicutes from the small intestine to the large intestine decreased, whereas the relative abundance of Proteobacteria increased (Figure 5F).

Compared with the NS group, antibiotics could reduce the abundance of intestinal microbiota in the small intestine, large intestine, and feces (Figure 5G). The number of observed microbiota species decreased, and the difference in the number of small and large intestine species decreased (Figure 5H). At the phylum level, antibiotics increased the relative abundance of microbiota in the small intestine (duodenum, jejunum, and colon) and increased the proportion of non-dominant microbiota in the NS group (Figure 5I). In the cluster heat map, each part of the large intestine (cecum, colon, and ileum) is similar. On the contrary, each part of the small intestine (duodenum, jejunum, and colon) is very different, and each has its own unique composition of intestinal microbiota (Figure 5J).

Compared with the NS group, the antibiotic resistant bacteria group, and the antibiotic group, the antibiotic resistant bacteria could resist the effect of antibiotics on the reduced abundance of intestinal microbiota in the antibiotic resistant bacteria + antibiotic group (Figure 5K). Resistance to antibiotics decreases the number of microbial species and increases the difference in the number of species in the small intestine and large intestines (Figure 5L). At the phylum level, resistant bacteria could increase the proportion of Bacteroidetes and Proteobacteria (Figure 5M). Moreover, antibiotics and donor bacteria have greater synergistic effects on the small intestine microbiota than on the large intestine (Figure 5N).

**Figure 6:**
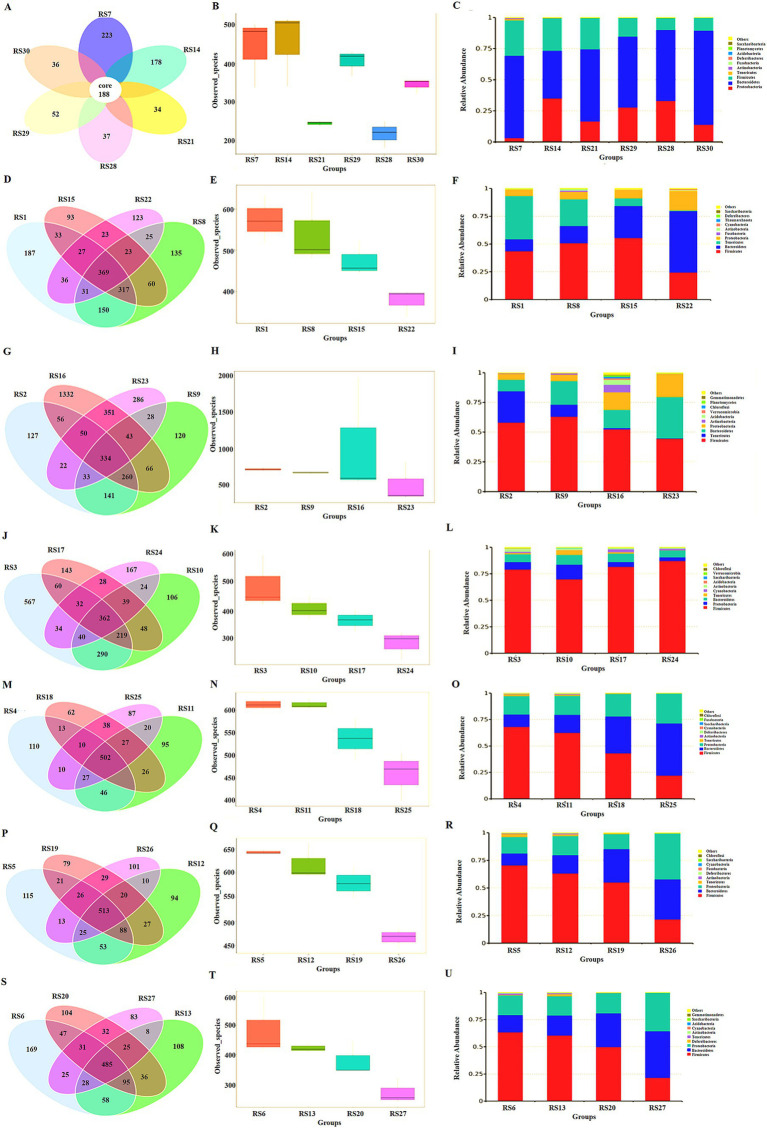
Microbiota analysis in different intestinal segments. **(A)** Abundance of intestinal microbiota in feces, RS7 NS group, RS14 resistant bacteria group, RS21 antibiotic group, RS29 resistant bacteria + low-dose antibiotic group, RS28 resistant bacteria + medium-dose antibiotic group, RS30 resistant bacteria + high-dose antibiotic group. **(B)** The number of species observed of intestinal microbiota in feces. **(C)** Intestinal microbiota analysis at the phylum level in feces. **(D)** Venn diagram of intestinal microbiota in the duodenum, RS1 NS group, RS8 resistant bacteria group, RS15 antibiotic group, and RS22 resistant bacteria + medium-dose antibiotic group. **(E)** Number of species observed of intestinal microbiota in duodenum. **(F)** Intestinal microbiota analysis in the duodenum at the phylum level. **(G)** Venn diagram of intestinal microbiota in the jejunum, RS2 NS group, RS9 resistant bacteria group, RS16 antibiotic group, and RS23 resistant bacteria + medium-dose antibiotic group. **(H)** Number of species observed of intestinal microbiota in the jejunum. **(I)** Intestinal microbiota analysis in the jejunum at the phylum level. **(J)** Venn diagram of intestinal microbiota in the ileum, RS3 NS group, RS10 resistant bacteria group, RS17 antibiotic group, and RS24 resistant bacteria + medium-dose antibiotic group. **(K)** Number of species observed of intestinal microbiota in the ileum. **(L)** Intestinal microbiota analysis in the ileum at the phylum level. **(M)** Venn diagram of intestinal microbiota in cecum, RS4 NS group, RS11 resistant bacteria group, RS18 antibiotic group, and RS25 resistant bacteria + medium-dose antibiotic group. **(N)** Number of species observed of intestinal microbiota in the cecum. **(O)** Intestinal microbiota analysis in the cecum at the phylum level. **(P)** Venn diagram of intestinal microbiota in the colon, RS5 NS group, RS12 resistant bacteria group, RS19 antibiotic group, and RS26 resistant bacteria + medium-dose antibiotic group. **(Q)** Number of species observed of intestinal microbiota in the colon. **(R)** Intestinal microbiota analysis in the colon at the phylum level. **(S)** Venn diagram of intestinal microbiota in the rectum, RS6 NS group, RS13 resistant bacteria group, RS20 antibiotic group, and RS27 resistant bacteria + medium-dose antibiotic group. **(T)** Number of species observed of intestinal microbiota in the rectum. **(U)** Intestinal microbiota analysis in the rectum at the phylum level.

In the resistant bacterial control group, the proportion of Proteobacteria was remarkably increased in the stool (Figure 6A), and foreign bacteria colonized the intestine and changed the structure of the stool microbiota (Figure 6B). Intestinal microbiota was primarily composed of Bacteroidetes, Firmicutes, Proteobacteria, and Apicutes. Bacteroidetes and Firmicutes were more inhibited by antibiotics, and Proteobacteria were more resistant to antibiotics (Figure 6C).

In the duodenum, antibiotics inhibited the species of the microbiota. The main components of intestinal microbiota were Firmicutes, Apicutes, Bacteroidetes, and Proteobacteria. Apicutes was evidently inhibited, and the proportion of Proteobacteria, Bacteroidetes, and Firmicutes was increased (Figures 6D–F).

In the jejunum, antibiotics inhibited the species and abundance of the microbiota. The main components of intestinal microbiota were Firmicutes, Apicutes, Bacteroidetes, and Proteobacteria. The proportion of Apicutes was decreased, and the proportion of Proteobacteria and Actinobacteria was increased (Figures 6G–I).

In the ileum, antibiotics reduced the number and complexity of the strains. The main components of intestinal microbiota were Firmicutes, Bacteroidetes, Proteobacteria, Actinobacteria and Anmicutes. Antibiotic resistant bacteria increased Proteobacteria and Bacteroidetes. Antibiotics increased the proportion of firmicutes and increased the proportion of Cyanobacteria (Figures 6J–L).

In the cecum, the resistant bacteria did not change the number of bacterial species in the cecum, and antibiotics inhibited the microbiota in this intestinal segment. The number of Firmicutes decreases, whereas that of Bacteroidetes increases. Antibiotics increased the proportion of Proteobacteria. Therefore, Proteobacteria might be the main receptor bacteria for ARGs in the intestine (Figures 6M–O).

In the colon, antibiotics inhibited the number of bacterial species. The combined action of antibiotic resistant bacteria and antibiotics decreased the proportion of posterior micutes and increased the proportion of Bacteroidetes. Consequently, the main components of this intestinal segment were Firmicutes, Proteobacteria, Bacteroidetes, and Apicutes in turn. The proportion of Proteobacteria in the antibiotic resistant bacteria control group did not significantly increase, indicating that the donor bacteria had not colonized in this part (Figures 6P–R).

In the rectum, antibiotics reduced the number of bacteria in the microbiota. The main components of this intestinal segment were Firmicutes, Proteobacteria and Bacteroidetes. The proportion of Proteobacteria in the antibiotic resistant bacteria in the control group did not change markedly, indicating that the ARG donor bacteria did not colonize the site (Figures 6S–U).

## Discussion

In this study, ARGs could achieve conjugative transfer through the plasmid RP4 or RK2, as well as location and diffusion in the intestinal tract of mice. Traditionally, the large intestine (colon and rectum) contains many types and large numbers of bacteria, which is the main location and transfer site of ARGs (Sylte et al., 2019; Rooney et al., 2019). However, the results of this study show that the small intestine (jejunum and ileum) has a greater relative microbiota abundance and more relative distribution of ARGs and donor bacteria, which is the true location and transfer area of ARGs. The results of flow cytometric screening indicated the conjugants of antibiotic resistant bacteria mainly existed in the small intestine, and the small intestine was the main site of conjugative transfer of ARGs. Although the results of fluorescence tracer imaging of small animals and flow cytometry showed that the number of resistant bacteria in the large intestine and feces was in the majority in absolute terms, it was in the minority relative to the total number of bacteria (Ding et al., 2020). The results of *16S rRNA* gene amplification and sequencing showed that the microbiota structure of the small intestine, large intestine, and feces were significantly different. From the small intestine to the large intestine, the abundance of bacteria decreased, whereas the number of bacteria increased. The ileum of the small intestine achieved a better combination of the abundance of bacteria and the number of bacteria, which was the main site for the transfer of ARGs.

The intestinal microorganisms in the small intestine (duodenum, jejunum, and ileum) had a stronger ability to acquire ARGs than those in the large intestine (cecum, colon, and rectum), because of the different compositions of intestinal microbiota in different segments of the intestine and their stronger ability to conjugative transfer. The results of the study showed that Proteobacteria and Bacteroidetes likely obtained resistance genes, and it’s consistent with the previous report (Fu, 2017; Ding et al., 2020). The distribution of microorganisms in different segments of the intestine is affected by oxygen, pH, and nutritional status. Proteobacteria in food, drinking water, or the environment, such as *E. coli*, enter the small intestine through the mouth, esophagus, and stomach, and an appropriate amount of oxygen is found in the small intestine, which is conducive to the active metabolism of bacteria and further conducive to the digestion and absorption of nutrients, which corresponds to the main function of the small intestine. Antibiotic resistant bacteria and antibiotics further increase the proportion of Proteobacteria, which is conducive to the digestion and absorption of food. The large intestine has less oxygen, slow cell metabolism, and more Firmicutes, which is related to their phenotypic resistance to antibiotics, rather than antibiotic resistance through the transfer of resistance genes. Although a large number of antibiotic resistant bacteria accumulated in the large intestine, the relative proportion of antibiotic resistant bacteria was lower than that in the small intestine. Bacteroidetes are also important receptor bacteria for ARGs in the gut. The microorganisms in this phylum, such as mycobacterium, are pathogenic bacteria under certain conditions, so such microorganisms increase the risk of disease in animals while obtaining ARGs (Qin et al., 2010; Ding et al., 2020; Ramakrishna and Patankar, 2023).

## Conclusion

In this study, the effect of the conjugative transfer of ARGs mediated by plasmids on the microecology of different intestinal segments was observed. The ileum of the small intestine was the main site for ARG transfer. Proteobacteria and Bacteroidetes were the main receptor bacteria of ARGs, which can easily obtain antibiotic resistance.

## Data Availability

The original contributions presented in the study are included in the article/Supplementary material, further inquiries can be directed to the corresponding authors.
