# Supplementary material for: Effect of conjugative transfer of antibiotic resistance genes mediated by plasmids on the microecology of different intestinal segments
Source: Front Microbiol. 2024 Dec 23;15:1504659. doi: 10.3389/fmicb.2024.1504659 (PMC11700969; doi:10.3389/fmicb.2024.1504659)
Supplement: Supplementary file 1 [file Data_Sheet_1.doc]

**Supplementary Information Appendix**


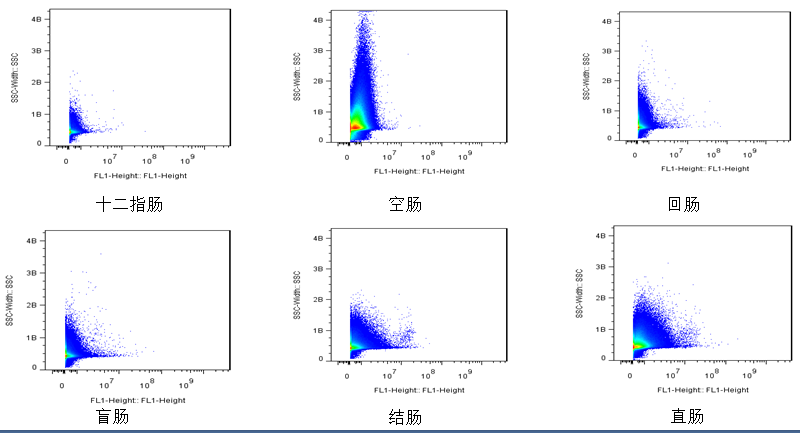


1 2 3


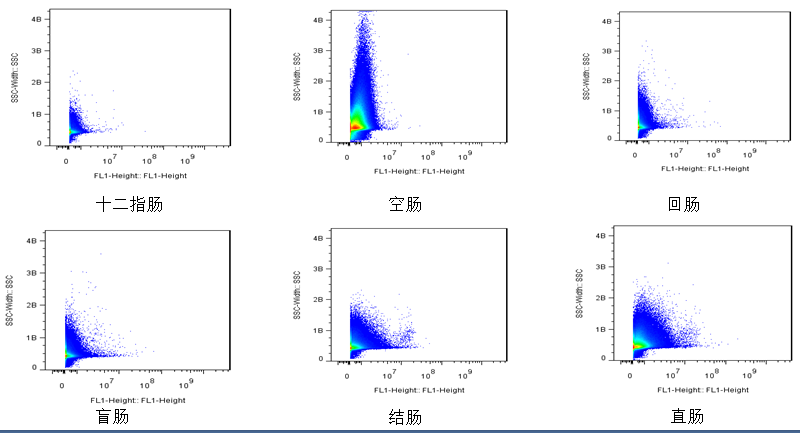


4 5 6

Figure S1 Flow cytometry screening of intestinal bacteria in NS control group

1 duodenum, 2 jejunum, 3 ileum, 4 cecum, 5 colon, 6 rectum


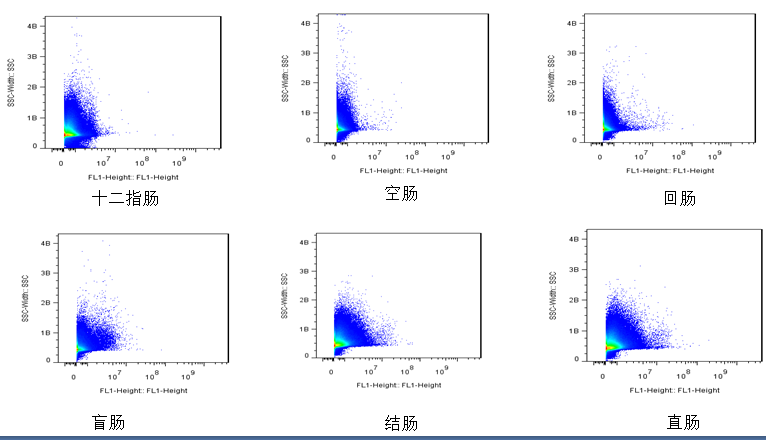


1 2 3


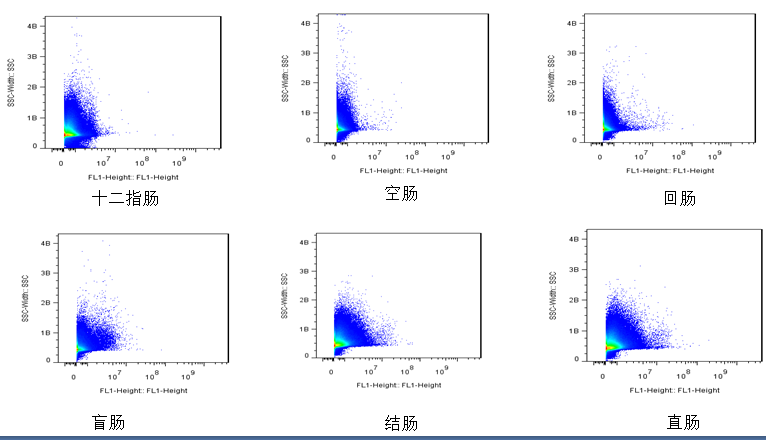


4 5 6

Figure S2 Flow cytometric screening of intestinal bacteria in K12 (RK2: EGFP) group

1 duodenum, 2 jejunum, 3 ileum, 4 cecum, 5 colon, 6 rectum
